# Heterogeneous CD8^+^ T Cell Migration in the Lymph Node in the Absence of Inflammation Revealed by Quantitative Migration Analysis

**DOI:** 10.1371/journal.pcbi.1004058

**Published:** 2015-02-18

**Authors:** Edward J. Banigan, Tajie H. Harris, David A. Christian, Christopher A. Hunter, Andrea J. Liu

**Affiliations:** 1 Department of Physics and Astronomy, School of Arts and Sciences, University of Pennsylvania, Philadelphia, Philadelphia, United States of America; 2 Department of Physics and Astronomy, Northwestern University, Evanston, Illinois, United States of America; 3 Department of Pathobiology, School of Veterinary Medicine, University of Pennsylvania, Philadelphia, Philadelphia, United States of America; 4 Department of Neuroscience, School of Medicine, University of Virginia, Charlottesville, Virginia, United States of America; Imperial College London, UNITED KINGDOM

## Abstract

The three-dimensional positions of immune cells can be tracked in live tissues precisely as a function of time using two-photon microscopy. However, standard methods of analysis used in the field and experimental artifacts can bias interpretations and obscure important aspects of cell migration such as directional migration and non-Brownian walk statistics. Therefore, methods were developed for minimizing drift artifacts, identifying directional and anisotropic (asymmetric) migration, and classifying cell migration statistics. These methods were applied to describe the migration statistics of CD8+ T cells in uninflamed lymph nodes. Contrary to current models, CD8+ T cell statistics are not well described by a straightforward persistent random walk model. Instead, a model in which one population of cells moves via Brownian-like motion and another population follows variable persistent random walks with noise reproduces multiple statistical measures of CD8+ T cell migration in the lymph node in the absence of inflammation.

## Author Summary

Migration is fundamental to immune cell function, and accurate quantitative methods are crucial for analyzing and interpreting migration statistics. However, existing methods of analysis cannot uniquely describe cell behavior and suffer from various limitations. This complicates efforts to address questions such as to what extent chemotactic signals direct cellular behaviors and how random migration of many cells leads to coordinated immune response. We therefore develop methods that provide a complete description of migration with a minimum of assumptions and describe specific quantities for characterizing directional motion. Using numerical simulations and experimental data, we evaluate these measures and discuss methods to minimize the effects of experimental artifacts. These methodologies may be applied to various migrating cells or organisms. We apply our approach to an important model system, T cells migrating in lymph node. Surprisingly, we find that the canonical Brownian-walker-like model does not accurately describe migration. Instead, we find that T cells move heterogeneously and are described by a two-population model of persistent and diffusive random walkers. This model is completely different from the generalized Lévy walk model that describes activated T cells in brains infected with *Toxoplasma gondii*, indicating that T cells exhibit distinct migration statistics in different tissues.

This is a *PLOS Computational Biology* Methods paper.

## Introduction

A primary challenge of immunological imaging experimentation is to understand the nature of cell migration statistics, and the role that these statistics play in immune function. Over the last decade, two-photon microscopy has transformed the understanding of the role of cell migration in the immune response [1–3]. However, although improved statistical approaches are still being developed [4–6], many existing methods for analyzing migration statistics are susceptible to experimental artifacts that can lead to inaccurate conclusions about leukocyte behavior. Similar questions arise in analyzing the migration of organisms ranging from bacteria [7] to vultures [8] and human hunter-gatherers [9]. Migration tracks can be directional or random and can be characterized by a bewildering array of models. This poses the question of how best to analyze migration tracks in an unbiased fashion, given experimental data that is often gathered in a limited field of view over a short period of time.

Many immune functions are thought to be directed by chemotactic signals, and directional migration has been observed in numerous cases, such as neutrophil response to sterile inflammation [10], migration of positively selected T cells in the thymus [11], and T cell priming by dendritic cells in lymph nodes [12, 13]. While the directional bias in these studies is clear, they use measures of directionality that can be susceptible to experimental artifacts. These issues range from technical constraints such as the finite imaging field and global drift to the intrinsic limitations of widely-used quantities such as the meandering index and motility coefficient [2, 4, 5, 14, 15]. Such artifacts can affect quantitative analyses and can even lead to inaccurate qualitative conclusions in cases where directional motility is subtle.

Immune cell migration also has a stochastic component [1, 3, 6, 16–18]. Commonly used random walk analyses [14, 15] assume that cells obey Brownian statistics. In several cases, however, it has been argued that cells migrate via persistent random walks [5, 16, 19–22] or even exhibit Lévy behavior [6, 23]. Despite this knowledge, many analyses of random motion implicitly assume that the statistics are described by Brownian walks even at short time scales, by assuming that the mean-squared displacement increases linearly in time and extracting a motility coefficient [14, 15]. The accurate identification of the persistence time for persistent random walks [5, 16, 19–22], or of more exotic forms of migration statistics such as Lévy behavior of migrating microglia [23] or of CD8^+^ T cells in *Toxoplasma gondii*-infected mouse brains [6], requires a description that goes beyond use of the mean-squared displacement as a distinctive identifier of migration statistics.

A more complete and accurate description of migration statistics requires methods capable of detecting subtle directional bias that can also handle more general forms of random walks, given experimental data gathered over rather short time periods. Furthermore, in order to investigate the correlation between cell migratory behavior and immune function, it is necessary to develop a description that rigorously characterizes both stochastic and directional migration without any initial assumptions regarding the location of possible targets. Current methodologies are of limited use in achieving these goals. Here, we describe a set of analytical and computational methods that can be used to identify various types of directional, anisotropic (asymmetric), and stochastic migration. These methods can be applied to any type of motile organism or cell. In order to demonstrate the practical implementation of the methods, we apply them to the migration of CD8^+^ T cells in uninflamed mouse lymph nodes. Unexpectedly, this system is well-described by a model containing two populations of T cells, in which one population obeys Brownian statistics and the other population migrates via heterogeneous, persistent random walks. While this model shares similarities with previous persistent random walk and run/pause models [5, 16, 19–22], we show that it reproduces several key statistical measures beyond the mean-squared displacement alone. Our results show that CD8^+^ T cells in uninflamed mouse lymph nodes migrate differently from those in the brains of mice chronically infected with *Toxoplasma gondii*.

## Results

In the analysis that follows, the migration statistics of CD8^+^ T cells in uninflamed lymph nodes are described using green fluorescent protein (GFP)-expressing OT-I T cells that were transferred to C57BL/6 mice as described in [24] and the Methods section. During the period of 16–24 hours after transfer, T cells were imaged in excised lymph nodes in a 500 *μ*m × 500 *μ*m × 68 *μ*m volume. In the following subsections, there are frequent references to numerical simulations of various walk models that were conducted to either illustrate our points or to describe our experimental data. We distinguish between results from simulations and from experiments by always identifying the walk model used in the simulation.

### Identifying directional motion

A pivotal question for many studies of immune cells is whether they migrate and respond to signals with specific directional motion or biases [2, 3, 14, 15]. More generally, anisotropic motion — motion that is not statistically identical in all directions — can indicate a directional bias, such as chemotaxis or chemokinesis due to a chemical gradient, or to an asymmetric feature of a particular direction, such as confinement. However, since even isotropic trajectories appear directed on short enough time scales, and, conversely, directed tracks typically contain a stochastic component, discerning directionality and anisotropy is not a simple task [15, 16].

The most commonly used method for identifying directional motion currently consists of plotting cell tracks with their starting points translated to the origin, measuring the MSD, calculating the meandering index, and measuring the mean displacement vector [5, 10–13, 15, 18]. However, as discussed in the following sections, these methods are only sensitive to obvious directionality, suffer from several experimental artifacts, and depend quantitatively on details of the experimental set up. Of these methods, even the quantitative tests are only capable of identifying global drift. Thus, they cannot detect other types of anisotropic motion, such as directed motion towards a single target (or scattered collection of targets) or Brownian-walk-like motion with different motility coefficients for different spatial directions. Other methods for identifying motion in a specific direction, such as measuring the component of velocity in that direction or angle of motion with respect to a target, require prior knowledge of the existence of a target or special direction [15].

Because of these issues, we developed and tested several techniques to detect and determine the amount and type of anisotropy. These methods are sensitive to small anisotropies and do not rely on having prior knowledge about the directional motion.

#### Correcting for time-dependent overall drift

A major problem in live tissue experiments is uncontrolled global drift due to factors related to sample preparation, including fluid flow, sample movement, and respiration [2, 4, 15]. This background drift introduces experimental artifacts that may lead one to overestimate cell mobility or incorrectly conclude that there is globally directed migration. It has previously been proposed that this drift can be measured by monitoring static structures within the tissue [15]. However, this method cannot account for issues such as sample movement and animal respiration. Instead, drift can be measured by monitoring passive auto-fluorescent particles, which should be nearly immobile. Any motion of these particles should be due to either the background drift or random Brownian-like motion. This phenomenon can be used to quantify drift and correct raw data sets which might have been otherwise unusable.

**Figure 1:**
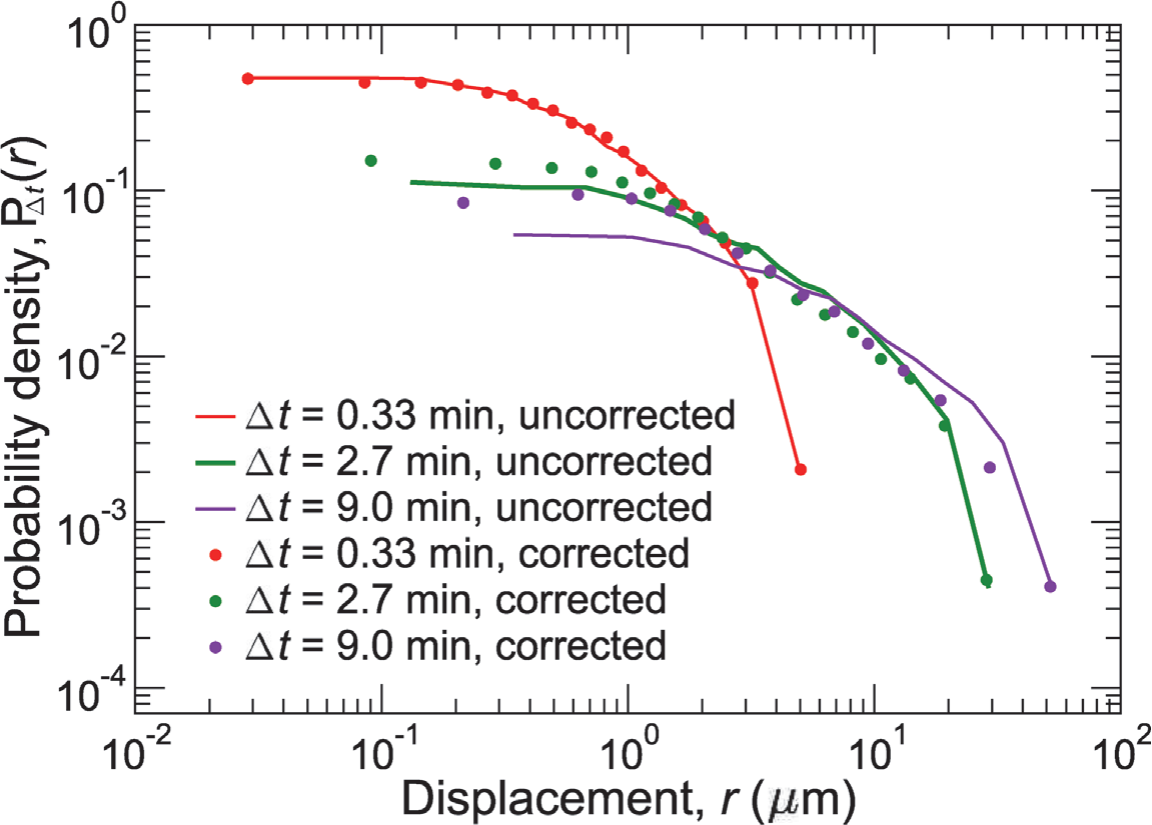
Correcting for drift. Probability distributions, *P*(*r*(*t*)), of displacements, *r*(*t*), at various times, *t*, are distorted by global drift (lines). To remove drift, we calculate adjusted probability distributions (circles) by removing the average motion of passive autofluorescent particles. The adjusted and unadjusted probability distributions differ significantly, especially at late times.

We calculate a single average trajectory (position as a function of time) from several (typically four, although more could be used) tracked particles in order to minimize the contribution of the random part of their motion to the estimate of global drift. These particles are assumed to move, on average, only as a result of global drift. The total contribution of global drift to cell migration at a given time is thus given by the average passive particle displacement at that time. By subtracting the passive particle displacement from the measured cell displacements, it is possible to obtain drift-corrected cell trajectories and displacements. The actual cell trajectories are more accurately given by these adjusted trajectories. Overall, this correction can lead to a significant shift in the computed trajectories of cells and overall migration statistics. This difference is shown, for example, by the histograms of unadjusted cell displacements (circles) and drift-corrected cell displacements (lines) for the T cells in Fig. 1. Although the drift correction, which is of order μm/min, is small over short observation periods (red in Fig. 1), it noticeably shifts the distributions after several minutes (green and purple). Similarly, other quantities describing cell migration are affected by the drift. All further analyses are performed on these drift-corrected data.

#### Testing for global directionality

Once corrections for global drift have been estimated, we look for directionality in the migration tracks by measuring the directional order parameter, *ϕ*, which has been used to describe the collective motion of animals [25, 26]. *ϕ* is calculated by computing the average of the normalized velocity vectors (whose components can take on positive or negative values), $\vec{u}=\langle \vec{v}/|\vec{v}|\rangle$ (where $\vec{v}$ is the velocity vector) and measuring the magnitude of the resulting vector, so that $\phi =|\vec{u}|$. If there is a global drift or bias in one direction, there should be a non-zero average velocity and a non-zero value of *ϕ*.

The order parameter *ϕ* is complementary to the mean velocity (or displacement) vector, $\left\langle \vec{v}\right\rangle$ ($\langle \vec{r}\rangle =\langle \vec{v}\rangle \Delta t$), because *ϕ* measures only angular direction. In some cases, this may be advantageous since variability in cell speeds contributes an additional component to the error in measuring the velocity vector $\left\langle \vec{v}\right\rangle$, and may obscure directionality. Furthermore, the magnitudes of cell velocities effectively weight some cells more heavily in directionality measurements using $\left\langle \vec{v}\right\rangle$; for directionality measurements with *ϕ*, all cells are weighted equally in the average. In addition, the mean velocity vector may not clearly detect drift along arbitrary combinations of the *x*, *y*, and *z* axes. Nonetheless, the mean velocity vector remains a useful quantity, since it is a speed-weighted average, and could highlight interesting features that the order parameter neglects. Since the utility of $\left\langle \vec{v}\right\rangle$ has already been demonstrated [5, 11], we present diagnostic results only for the directional order parameter, *ϕ*, below.

Since the contribution of diffusion may dominate over directed motion until long observation times [5, 27], measurements of *ϕ* may not be sensitive enough to detect biased motion in cell displacements that occur between just two imaging frames. However, the sensitivity can be amplified by measuring average velocities over a longer time segment rather than “instantaneous” velocity estimated by cellular displacements between adjacent time frames. However, since the duration of the experiment can be broken down into fewer long time segments than short time segments, the statistical error is higher for longer time segments; in addition, data from cells that leave the field of view in less time than the long time segment must be discarded, which can bias data (this issue is described in detail in the section “Analyzing displacement data”). One must therefore choose the length of the time segment to balance these considerations.

**Figure 2:**
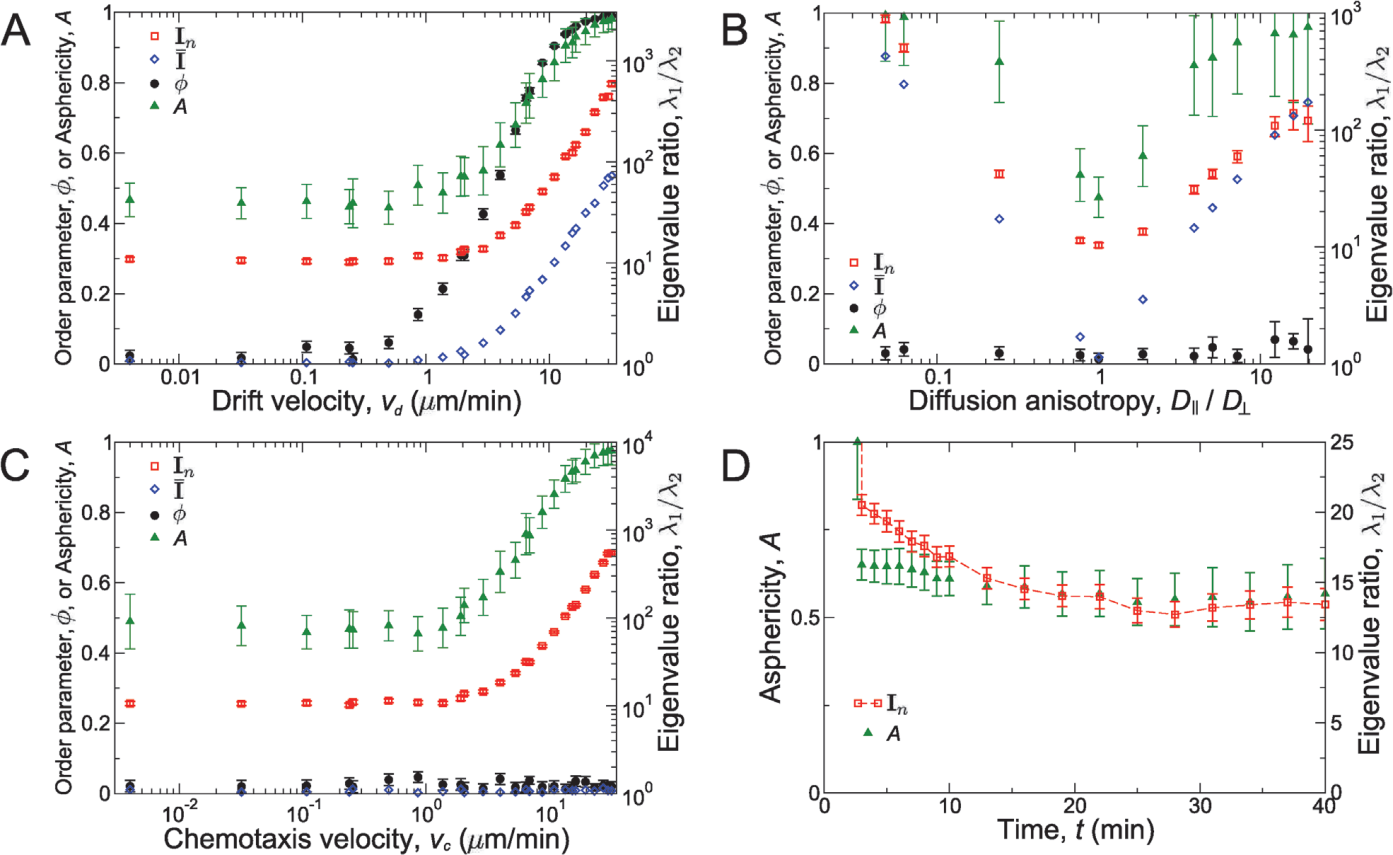
Testing measures of anisotropy and directionality. Measures of anisotropy as a function of (A) global drift velocity *v*_*d*_, (B) ratio, *D*_∥_/*D*_⊥_, of motility coefficients in different directions, and (C) chemotaxis velocity, *v*_*c*_, towards a central point. In general, when the anisotropy is small (*v*_*d*_ ≈ 0, *v*_*c*_ ≈ 0, and *D*_∥_/*D*_⊥_ ≈ 1), the anisotropy measures are small too. The directional order parameter, *ϕ*, measured with non-overlapping time intervals of 5 minutes, (solid black circles, left axes), is non-zero for *v*_*d*_ ≳ 1 *μ*m/min (panel (A)) but is zero or very small otherwise (panels (B) and (C)). The ratios of eigenvalues, *λ*_1_/*λ*_2_ (open symbols, right axes), averaged over all individual track inertia tensors, **I**_*n*_ (open red squares) or derived from the average of inertia tensor, $\bar{I}$ (open blue diamonds), and asphericity (solid green triangles, left axes) are non-zero for sufficiently large *v*_*d*_ (panel (A)) and *D*_∥_/*D*_⊥_ that deviates sufficiently from 1 (panel (B)). Only the average eigenvalue ratio over individual track inertia tensors and asphericity are non-zero for $v_{c}≳1\;\mu \text{m/min}$ (panel (C)). (D) The ratio, 〈*λ*_1_〉/〈*λ*_2_〉, of average eigenvalues of individual track moment of inertia tensors (open red squares, right axes) and asphericity, *A* (solid green triangles, left axes), for an isotropic persistent random walk is large at early times, but decays as a function of time. The red dashed line is a guide to the eye. Error bars show standard error of the mean (SEM).

To demonstrate how to use the order parameter, we measure it for a series of numerical simulations of 5000 random walkers (simulated cells). The walkers diffuse with motility coefficient *D* = 30 *μ*m^2^/min, similar to T cells in various experiments [1, 5, 28–30] and are biased to move in the +*x* direction with speed $\left|\vec{v}\right|$. As in our experiments, we track the simulated walkers in a 500 *μ*m × 500 *μ*m × 68 *μ*m imaging field for 10 minutes (thus we track the approximately 1000 simulated walkers that remain in the field for at least two frames). The solid black circles in Fig. 2A show that the order parameter detects global drift as small as fractions of a micron per minute. For large bias, $\left|\vec{v}\right|$, the directional order parameter *ϕ* is large, indicating that many cellular movements have the same directionality. However, as the drift velocity decreases, the simulated walkers become more like pure Brownian walkers, and thus, *ϕ* decreases toward zero.

We now calculate *ϕ* for real data for CD8^+^ T cell tracks in the uninflamed lymph node. We find that *ϕ* for a given imaging experiment is typically more than a standard error of the mean away from zero, but nonetheless lies within a 95% confidence interval of zero for six of eight imaging series. Note that compared to data that has not been adjusted for overall drift, *ϕ* is decreased, on average, by about 50%. As a means of determining whether the detected bias is significant, we analyzed cell migration data after removing the components of motion directed along $\vec{u}$. We verified that removing the cellular motions along this direction did not significantly alter the measures of migration statistics of the full data set described below. Furthermore, the average magnitude, *V* = 0.80 ± 0.68 *μ*m/min, of the mean velocity vector is small. Thus, the analysis of *ϕ* suggests that there is little global directional bias in CD8^+^ T cell tracks in the uninflamed lymph node over the time and volumes imaged.

#### Calculating the moment of inertia tensor for cell tracks to detect other anisotropies

While the directional order parameter *ϕ* can reliably detect preferential motion in a given direction, it cannot detect other types of anisotropy. For instance, *ϕ* does not indicate differences in the motility coefficients in different directions (*e.g.*, *ϕ* = 0 for *D*_*x*_ ≠ *D*_*y*_; solid black circles in Fig. 2B) or directed motion towards a target (solid black circles in *ϕ* = 0 for a single target at the center of the image; Fig. 2C).

To reliably identify all types of anisotropy, we introduced the moment of inertia tensor, **I**, which can be used to detect various anisotropies in cellular migration [6, 31, 32]. **I** is a *d* × *d* matrix quantifying geometrical information about the *d*-dimensional spatial positions visited by the cells (see Methods section for details). In most imaging studies, cellular trajectories are artificially compressed in the *z* direction due to limited depth of view. Thus, we restrict the analysis to *x* and *y* positional information and construct the two-dimensional moment of inertia tensor. Nonetheless, this procedure can easily be extended to analyze the three-dimensional moment of inertia tensor. By calculating the characteristic values (eigenvalues), *λ*_1_ and *λ*_2_, of the matrix **I** (see Methods section and *e.g.*, refs. [31, 32]), it can be determined whether cells prefer to move along a particular spatial axis (*x*, *y*, or some combination). If the characteristic values are equal (*λ*_1_ = *λ*_2_), cell migration is isotropic — every spatial direction is the same. However, if one characteristic value is larger than the other (*λ*_1_ > *λ*_2_; by convention, *λ*_1_ ≥ *λ*_2_), this indicates greater cell migration along a particular axis, which is given by the corresponding eigenvector (see [31, 32]). The ratio, *λ*_1_/*λ*_2_, quantifies the amount of anisotropy. As we describe below, an average moment of inertia tensor, $\bar{I}$, can be calculated for average cell motions, and a set of individual moment of inertia tensors, **I**_*n*_, can be calculated for the motions of individual cells. These two calculations provide complementary information about migration statistics.

The average moment of inertia tensor, $\bar{I}$, over all cellular motions quantifies the geometry of the average of all cell trajectories. By calculating the eigenvalues of this quantity, we can determine whether there is a particular axis along which all cells, on average, prefer to move. Similar to the directional order parameter *ϕ*, the ratio, ${\bar{\lambda }}_{1}/{\bar{\lambda }}_{2}$, of eigenvalues of $\bar{I}$ can be used to detect global drift in numerical simulations (open blue diamonds in Fig. 2A), albeit with slightly lower sensitivity. However, unlike *ϕ*, the ratio ${\bar{\lambda }}_{1}/{\bar{\lambda }}_{2}$ can be used to detect differences in motility coefficients of diffusive motions in different directions. To show this, we simulated walkers where diffusion in one direction occurs with motility coefficient *D*_∥_, which is varied (*e.g.*, we choose the *x* direction). Diffusion in directions perpendicular to the first (the *y* and *z* directions) occurs with a fixed motility coefficient *D*_⊥_ = 10 *μ*m^2^/min. As shown by the open blue diamonds in Fig. 2B, the ratio ${\bar{\lambda }}_{1}/{\bar{\lambda }}_{2}$ grows large for *D*_∥_ noticeably different from *D*_⊥_ (*D*_∥_/*D*_⊥_ ≠ 1).

Neither the *average* inertia tensor $\bar{I}$ nor the directional order parameter *ϕ* can be used to identify motion toward a central target (open blue diamonds and solid black circles, respectively, in Fig. 2C). However, by calculating the moment of inertia tensors for individual tracks, **I**_*n*_, and averaging their eigenvalues to find 〈*λ*_1_〉 and 〈*λ*_2_〉, we can identify motion toward a central target. The open red squares in Fig. 2C show that 〈*λ*_1_〉/〈*λ*_2_〉 can be used to detect chemotaxis toward a central target at velocities of order *μ*m/min or larger. Although Fig. 2C demonstrates the efficacy of using **I**_*n*_ to detect motion towards a known central target, the set of individual track moment of inertia tensors can be used to detect chemotactic motion toward a target at an arbitrary location. Moreover, we emphasize that **I**_*n*_ (as well as the other quantities discussed in this section) can detect anisotropic motion without prior knowledge of the target location or even the existence of a special direction. As shown in Fig. 2A-B, this ratio can also be used to detect other types of anisotropy.

Thus, in conjunction with the order parameter *ϕ*, the moment of inertia tensor analysis can detect and distinguish between three kinds of anisotropy — global drift, direction-dependent diffusion, and motion toward a single point. These quantities can be used to compare different migration data sets, and the distributions of track eigenvalues, *λ*_1_ and *λ*_2_, provide a constraint for model selection. Table 1 lists the ability of each of these quantities to identify the three types of anisotropy discussed.

**Table 1:** Summary of measures of directionality. The four quantities described in section “Identifying directional motion” can be used to identify several types of anisotropy. Together, they can be used to identify each of the three types of anisotropy discussed.

Quantity	Global drift ($|\vec{v}|\neq 0$)	Anisotropic diffusion (*D*_⊥_ ≠ *D*_∥_)	Motion towards a central point ($\left({\vec{v}}_{c}\neq 0\right)$)
*ϕ*	Yes	No	No
$\bar{I}$	Yes	Yes	No
**I**_*n*_	Yes	Yes	Yes
*A*	Yes	Yes	Yes

From the moment of inertia tensor and eigenvalues, we calculate the mean asphericity, *A*, of the tracks [33]. This quantity, defined as $A=\frac{\langle (1/2){\left(\lambda_{1}+\lambda_{2}\right)}^{2}-2\lambda_{1}\lambda_{2})\rangle }{\langle (1/2){\left(\lambda_{1}+\lambda_{2}\right)}^{2}\rangle }$ in two dimensions, measures the deviation of the tracks from spherical symmetry; asphericity ranges from *A* = 0 if tracks are perfectly spherical (circular in two dimensions) to *A* = 1 for totally elongated tracks. While asphericity, *A*, does not provide detailed information about the different principal axes and is less precise than the other described measures (*e.g.*, notice the larger error bars in Fig. 2), it does allow the comparison of the measured asphericity to the theoretical value of *A* = 4/7 ( ≈ 0.57) for a two-dimensional random walk [33].

To determine whether CD8^+^ T cells in lymph nodes in the absence of inflammation exhibit directional motion, we apply this analysis to the experimental data. The asphericity, *A* = 0.73 ± 0.28, and the ratio of average individual track eigenvalues, 〈*λ*_1_〉/〈*λ*_2_〉 = 28.6 ± 7.1, are both larger than expected for an isotropic random walk. Nonetheless, 〈*λ*_1_〉/〈*λ*_2_〉 is not very large (compare to values at small anisotropy in Fig. 2A-C) and due to the large error in *A*, asphericity alone does not provide definitive information. The eigenvalues, ${\bar{\lambda }}_{1}$ and ${\bar{\lambda }}_{2}$, of the total inertia tensor, $\bar{I}$, for each movie are typically the same order of magnitude, but differ due to statistical noise in the cell tracks.

The fact that the observed ratio, 〈*λ*_1_〉/〈*λ*_2_〉, and asphericity, *A*, are larger than expected for Brownian random walks might be perceived as indicative of anisotropic walks. But, it is important to note that (1) the observed walks are necessarily rather short due to cells leaving the field of view through the shallow *z* direction and (2) the walks may be isotropic and random but not Brownian. Both of these factors will give rise to apparent anisotropy at short time scales. Moreover, nearly all migrating cells move unidirectionally on short enough time scales [15, 16], and some move persistently for several minutes or longer [5, 6, 19, 21].

To demonstrate this principle, we simulate 5000 persistent random walkers with representative values of motility coefficient, *D* = 29 *μ*m/min, and persistence time, *t*_*p*_ = 81 s [1, 5, 19, 21, 28–30] in a finite volume (note that the values of *D* and *t*_*p*_ were calculated in a simulated infinite field of view). Although the model is isotropic so that cells do not have a preferred direction, many individual cell tracks are nearly unidirectional on the experimental time scale. This is reflected by the measurements of 〈*λ*_1_〉/〈*λ*_2_〉 and *A*, which are large for short observation periods, but decreases to smaller values for simulated experiments lasting more than 15 minutes (Fig. 2D). This example highlights the difficulty in interpreting the described quantities even if they indicate that anisotropic motion is present. Before concluding that cell migrate directionally, one must rule out alternative sources of apparent directionality, such as the limited duration of the experiment. For the case of CD8^+^ T cells, our simulations suggest that the observed values of 〈*λ*_1_〉/〈*λ*_2_〉 and *A* are not the result of directional migration. Rather, as described below, they are expected from our non-directional migration model with a limited experimental observation time.

**Figure 3:**
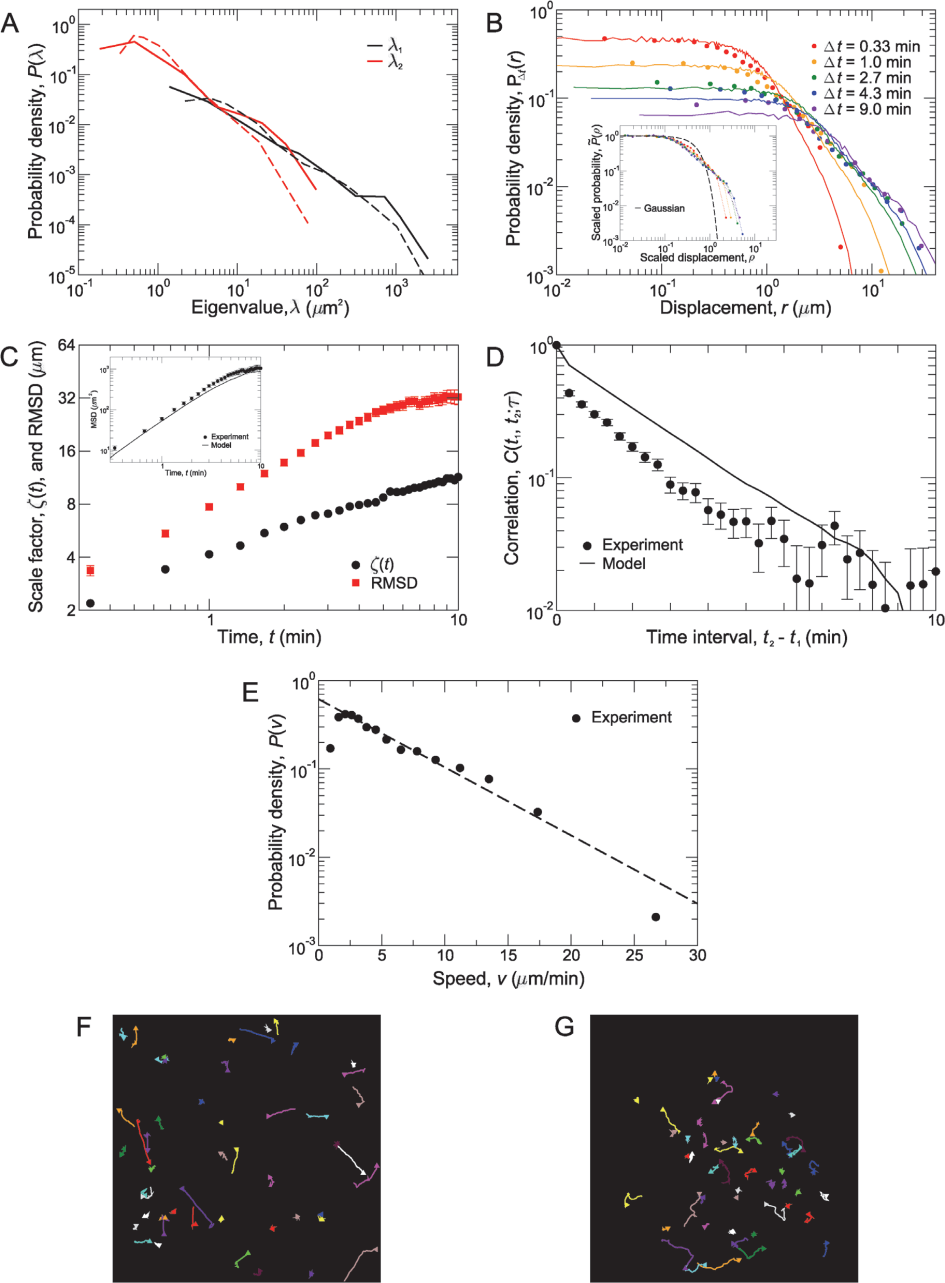
Analysis of T cell migration. (A) The experimentally observed distributions of eigenvalues, *λ*_1_ and *λ*_2_, for individual track moment of inertia tensors, **I**_*n*_ (black and red solid lines, respectively). The distributions of track eigenvalues for the two populations walk model agree well (dashed lines) with experiment. (B) Drift-corrected probability distributions, *P*(*r*(*t*)), of T cell displacements, *r*(*t*), at various times, *t* (circles) and probability distributions of simulated cell displacements (solid lines). *Inset*: The scaled distributions, $\tilde{P}(\rho )$, of *ρ* = *r*/*ζ*, of T cell displacements fall on one curve (circles) at small *ρ*. This is clearly not a single Gaussian distribution (dashed line). Colored dotted lines in the inset are a guide for the eye to more clearly see systematic deviations from perfect scaling collapse at large *ρ*. (C) The scaling factor, *ζ*(*t*) (black circles), and RMSD (red squares) as a function of time. Initially, these grow faster than $\sqrt{t}$, but at intermediate to late times, they approach the scalings expected for long-time diffusive behavior. The slopes are different on the log-log plot, indicating that *ζ*(*t*) and the RMSD have different time dependences and are not proportional to each other. *Inset*: The MSD of walkers in the model (line) agrees well with the experimentally observed MSD (circles). (D) Normalized correlations, *C*(*t*_1_, *t*_2_;*τ*), between the displacements $\vec{r}(t_{1},t_{1}+\tau )$ and $\vec{r}(t_{2},t_{2}+\tau )$ (which occur over the time intervals *t*_1_ < *t* < *t*_1_+τ and *t*_2_ < *t* < *t*_2_+τ, respectively), plotted as a function of *t*_2_ – *t*_1_. *C*(*t*_1_, *t*_2_;*τ*) for T cells (circles) initially drops sharply, and then decays as a straight line on the semi-logarithmic plot, indicating exponential decay. At long time intervals, *t*_2_ – *t*_1_, there are deviations from exponential decay. The model (line) agrees qualitatively. Although the magnitude of normalized correlations is greater, the correlations have the same functional dependence and decay with the same persistence time. Error bars in (C) and (D) show SEM. (E) Experimentally observed instantaneous speed distribution, *P*(*v*) (circles). This is estimated by the distance traveled over 20 s, the time between imaging frames. Dashed line is an exponential decay, $\propto e^{-v/v_{0}}$, where *v*_0_ = 5.63 μm/min. (F) Simulated trajectories for walkers in our model projected into 2D. (G) Experimentally observed trajectories of T cells in uninflamed lymph nodes.

Individually, each of these approaches have the ability to detect different types of anisotropic migration, but they must be used together in order to properly classify anisotropy. Note that once anisotropy is detected, experimental artifacts must be ruled out. This is most easily done within the context of a random walk model that fits the displacement data, as discussed below. One can then calculate the ratio 〈*λ*_1_〉/〈*λ*_2_〉 within the model for tracks of the time duration of the experiment, and compare the expected anisotropy from the model to the observed anisotropy from the experiment to see whether there is a significant difference. In the case of T cells in the lymph node in the absence of inflammation, we find that the distributions of *λ*_1_ and *λ*_2_ agree reasonably well with expectations for the isotropic non-Brownian random walk model presented below (Fig. 3A). Furthermore, the eigenvalues, ${\bar{\lambda }}_{1}$ and ${\bar{\lambda }}_{2}$, of the total inertia tensor, $\bar{I}$, for each imaging series are typically the same order of magnitude, but differ due to statistical noise in the cell tracks. Altogether, these measurements suggest that CD8^+^ T cell migration in lymph nodes during the steady state is globally isotropic on the time and length scales of the experiment performed.

#### Summary of directionality analysis

Traditionally, directionality is identified qualitatively by visualization of the cell tracks and quantitatively by measuring the average drift velocity, mean-squared displacement exponent, and comparing meandering indices across data sets. However, these methods are insensitive to some of the types of directionality described above. Moreover, quantities such as the MSD are biased by the finite imaging field, while others, such as the meandering index, are not robust across different experiments. In contrast, the methods we have described are more general and less affected by these limitations and they do not require prior knowledge of the target location or the axis of directional motion. When used together, the quantities described in this section can be used to identify several types of anisotropic motion; these results are summarized in Table 1. Moreover, the methods described directly measure the shape of cell tracks instead of a particular aspect of directional motion. Thus, these quantities, especially the individual track moment of inertia tensors, **I**_*n*_, can also describe non-directional motion and provide constraints for migration model selection. Using these measurements, we found that migration of CD8^+^ T cells in lymph nodes is globally isotropic on the time and length scales of this experiment.

### Analyzing displacement data

The mean-squared displacement, while straightforward to calculate, is highly susceptible to artifacts that can lead to misinterpretations of data. For example, typical imaging experiments can only visualize cells within a relatively small part of the space that cells can actually explore. Thus, as noted previously, cells may exit the field of view before the time series has ended, which can bias the analysis [5, 6, 15, 21, 30]. The magnitude of this effect can be estimated by calculating the mean time, 〈*t*_exit_〉, for a Brownian-random-walking cell to reach the boundary of the imaging field, which has a shortest dimension (typically, depth) of length *L* [27]:

$$\langle t_{\mathrm{exit}}\rangle =\frac{L^{2}}{12D},$$ (1)

where *D* is the diffusion (motility) coefficient. For typical values derived from multiple studies of T cell movement, *L* ≈ 40 *μ*m and *D* ≈ 30 *μ*m^2^/min (*e.g.*, refs. [1, 5, 6, 15, 28–30, 34]), 〈*t*_exit_〉 is just 4.4 minutes, with some cells exiting the field of view even more quickly.

This limitation of imaging has especially significant consequences for the mean-squared displacement (MSD). Since fast-moving cells tend to leave the imaging field more quickly than others, data at late times becomes biased toward slow-moving cells [5, 15, 21, 30]. This can distort the shape and magnitude of the MSD as a function of time. Furthermore, this issue plagues alternatives to the standard practice [5, 14, 15] of measuring the motility coefficient from the slope of the best-fit line to the MSD versus time curve. Since the standard motility coefficient method is inaccurate due to short-time directional persistence [5], one may try either fitting only the MSD at late times to a line or fitting the MSD with a function of both the motility coefficient, *D*, and persistence time, *t*_*p*_ [5, 21]. However, the first option exacerbates the finite imaging field problem, underestimating the diffusion coefficient by as much as 20% under simulated typical conditions. The second option is also inadequate because the fit parameters (motility coefficient and persistence time) are sensitive to the duration of the “early time” segment used. Alternatively, if long time segments are used, the fits converge on common parameters [5], but we have found in simulations that they underestimate the motility coefficient and persistence time by as much as 20% and 40%, respectively, due to cells exiting the imaging volume.

When considered together, these limitations have several practical implications. Since the mean time 〈*t*_exit_〉, for a cell to leave the imaging volume is typically a few minutes, and many cells exit earlier than 〈*t*_exit_〉, the MSD and quantities derived from the MSD are unreliable for rigorously assessing migration data. Counterintuitively, in a finite imaging field, measuring the MSD over longer time intervals can lead to erroneous conclusions rather than deeper insights. It should also be noted that the MSD does not uniquely specify an underlying model for random (or directional) motion [15, 35, 36]. Therefore, this measurement should only be used for qualitative comparisons between experiments with identical imaging dimensions or as a complementary consistency check for any proposed migration model.

Instead of focusing on the MSD, we rely on two main quantities to characterize the displacements: (1) the probability distribution, *P*_Δ*t*_(*r*), of cell displacements, *r*, as a function of the time interval Δ*t*, over which the displacement occurs and (2) the correlations, *C*(*t*_1_, *t*_2_;*τ*), between the displacements during one time interval *τ* starting at time *t*_1_, with displacements during a later time interval *τ*, starting at *t*_2_. Utilizing these quantities mitigates problems due to the finite field of view. Probability distributions, *P*_Δ*t*_(*r*), reveal displacements of various sizes and at all times, instead of focusing on large displacements at late times, which are the most profoundly impacted by the limited field of view. The correlation function typically reveals features such as persistence in the early-time data, while minimizing significant artifacts.

#### Displacement probability distributions

The probability distribution *P*_Δ*t*_(*r*) is the fundamental quantity underlying the migration statistics, and in principle, all of the statistical properties of migration, including directional biases, mean-squared displacement, and correlations, can be obtained from it. However, in order to determine the best way to construct *P*_Δ*t*_(*r*), it is useful to have an idea in advance of whether directional bias is present. For example, if there is overall motion in the *x*-direction, it is useful to focus on the *x*-displacements and to average over the *y*- and *z*-displacements, while if there is overall motion towards a central target, it is useful to focus on the distribution of radial displacements.

One can construct *P*_Δ*t*_(*r*) by plotting the histogram of cell displacements (or components of displacements), *r*, that occur over a time interval, Δ*t*, and normalizing by the total number, *N*, of displacements measured. As shown by animal migration studies, low resolution and linear or logarithmic binning methods can bias the data, leading to spurious results [37–39]. In order to avoid these artifacts and capture the true shape of *P*_Δ*t*_(*r*), we collect a large amount of data (several hundred tracks), place a fixed number of displacements in each bin, and set the bin widths according to the composition of each bin [6]. Specifically, we put *m* displacements in each bin, so that there will be *N*/*m* bins. The *i* ^th^ bin is centered at the average over all *m* displacements in that bin and the bin is assigned a weight of $W_{i}=1/(r_{\text{max}}^{\left(i\right)}-r_{\text{min}}^{\left(i\right)})$, where $r^{{\left(i\right)}_{\text{max}}}$ and $r^{{\left(i\right)}_{\text{min}}}$ are, respectively, the maximum and minimum displacements in the *i* ^th^ bin. The weighting procedure normalizes *P*_Δ*t*_(*r*), and more importantly, accounts for the variable width of bins, ensuring that the constructed *P*_Δ*t*_(*r*) accurately reflects the probability density function.

The effect of statistical errors can be evaluated by varying the number of points per bin and the number of bins in the histogram for both the complete set and subsets of the displacement data [6]. For a sufficiently large data set (typically ≳ 1000 displacements) errors in the positions and weights of the data points in the middle of *P*_Δ*t*_(*r*) are on the order of a few percent. Errors in bin locations and bin weights for, respectively, the smallest and largest displacement bins are significantly larger, but these errors do not affect the observed shape of *P*_Δ*t*_(*r*) and have a negligible effect on the ensuing analysis [6].

Statistical accuracy of the distribution can be improved by utilizing symmetries within the data. Since CD8^+^ T cell migration is isotropic, we binned the *x*-, *y*-, and *z*-components of displacements together for the displacement distributions in Fig. 3B (circles). For cases where T cell migration is not isotropic, *x*, *y*, and *z* displacements should be binned separately and displacements in the positive direction (+*x*, +*y*, or +*z*) should be distinguished from displacements in the negative (−*x*, −*y*, or −*z*) direction.

For example, consider *N* = 10,065 cell displacements occurring over all Δ*t* = 2.7 minute time intervals in the experiment. To construct the distribution of cell displacements over this time interval (green circles in Fig. 3B), we sorted the displacements in ascending order and constructed histograms with *m* = 700 displacements the first 14 bins and put the remaining 265 displacements in the final bin at large displacements, *r*. Each bin is centered at the average displacement for the bin, and its probability *P*, is *m*/*N* divided by the width of the bin (maximum minus minimum displacement in the bin). This process was repeated for Δ*t* = 0.33, 1.0, 2.7, 4.3, and 9.0 minutes using *m* = 850,800,700,600, and 330 counts per bin, respectively (Fig. 3B). These data serve as the foundation for the analysis of CD8^+^ T cell migration.

By constructing *P*_Δ*t*_(*r*) for multiple time intervals, Δ*t*, migration statistics can be characterized over the entire time course of the experiment. In Fig. 3B, histograms of cell motions over Δ*t* = 0.33,1.0,2.7,4.3, and 9.0 minutes are shown. As expected, the distributions broaden with increasing Δ*t*, because cells can move further over larger Δ*t*. However, the overall shape of *P*_Δ*t*_(*r*) at all observed times is broader than Gaussian.

To investigate the possibility of non-Gaussian scaling behavior, we scaled the probability distributions by a time-dependent scaling factor, *ζ*(Δ*t*), and plotted probability distributions, ${\tilde{P}}_{\Delta t}(\rho )=\zeta (\Delta t)P_{\Delta t}(r)$, of scaled displacements, *ρ* = *r*/*ζ*(Δ*t*) (inset to Fig. 3B). This factor is chosen for each time, Δ*t*, so that the rescaled probability distribution, ${\tilde{P}}_{\Delta t}(\rho )$, for that time, will lie on top of the other rescaled distributions. Often, this factor can be chosen by numerically fitting *P*_Δ*t*_(*r*) to a distribution function with a known scaling factor. However, for the data in Fig. 3B, we chose *ζ*(Δ*t*) so that the average ${\tilde{P}}_{\Delta t}(\rho )$ for the three smallest *ρ* in the histogram is equal to one. The qualitative behavior observed in the inset to Fig. 3B is not sensitive to the precise details of the procedure for choosing *ζ*(Δ*t*). In general, it may not be possible to perfectly collapse the probability distributions in this manner; thus, the scalability of the distributions provides useful information about the underlying migration statistics.

For CD8^+^ T cell migration statistics, the scaling factor, *ζ*(*t*), is not proportional to the root-MSD (RMSD), as would be expected for purely Gaussian behavior (Fig. 3C). Instead, *ζ*(*t*) (black circles in Fig. 3C) and the RMSD (red squares) have different time dependences. In addition, the distributions, ${\tilde{P}}_{\Delta t}(\rho )$, are not Gaussian (dashed line in the inset to Fig. 3B), indicating that migration is not Brownian-like over these time scales. However, although the collapsed distributions approximately fall on a single curve for small scaled displacements (ρ ≲ 1), deviations from the collapse at large values of *ρ* are systematic, so migration is also not Lévy-like on these time scales [6]. Intriguingly, the deviations from perfect collapse also indicate behavior that increasingly deviates from a single Gaussian distribution over time. From the central limit theorem, one would expect behavior to converge towards a Gaussian distribution at long times for a random walk whose step size is bounded, so the opposing trend apparent in the experimental data suggests that longer steps occur with greater frequency than expected.

#### Correlations between displacements

Four-point time correlation functions quantify persistence and memory in cell migration and provide an additional constraint for model selection [6, 40]. In particular, it is useful to measure the statistical relation between a quantity, such as cellular displacement, $\Delta \vec{r}(t_{1},t_{1}+\tau )$, between images taken at *t*_1_ and *t*_1_+*τ*, and another quantity, such as a later cellular displacement, $\Delta \vec{r}(t_{2},t_{2}+\tau )$ between times, *t*_2_ and *t*_2_+*τ*. Thus, in contrast to the meandering index [15], such correlations can precisely measure the distance and duration of straight runs before turns or stalls [40]. Moreover, unlike the meandering index, the overall shape of the correlation function does not depend on the duration of the experiment or the time interval, *τ*, between imaging frames (provided *τ* < *τ*_*p*_, where *τ*_*p*_ is the persistence time).

Correlation functions may also provide additional evidence or quantification of anisotropic or directional motion. This can clarify the interpretation of anisotropies identified by the measures in the section “Identifying directional motion.” For instance, the moment of inertia tensor might identify several distinct principal axes of motion. If this is due to different persistence times or lengths along these axes, then correlations between motions along these axes should differ.

Finally, it is in principle possible to investigate the response to different events, such as the introduction of a chemotactic signal, by investigating differences between correlation functions of cells at different times or locations.

To describe CD8^+^ T cell migration in the lymph node, we use the correlation function:

$$C\left(t_{1},t_{2};\tau \right)=\frac{\langle \Delta \vec{r}\left(t_{1},t_{1}+\tau \right)∙\Delta \vec{r}\left(t_{2},t_{2}+\tau \right)\rangle }{\langle {\left(\Delta \vec{r}\left(t,t+\tau \right)\right)}^{2}\rangle },$$ (2)

where $\Delta \vec{r}(t,t+\tau )$ is the vector change in position over the time interval starting at time *t* and ending at time *t*+*τ*. *C*(*t*_1_, *t*_2_;*τ*) measures the correlations between cell motions at different times, normalized by the average motion. The semi-logarithmic plot of the correlation function, *C*(*t*_1_, *t*_2_;*τ*), in Fig. 3D (circles), shows the decay of correlations over time. The correlation function decays primarily in a straight line on this scale, indicating that correlations typically drop off exponentially; thus, there is a well-defined apparent persistence time for *C*(*t*_1_, *t*_2_;*τ*), *t*_*p*_ ≈ 2 min (alternatively, a persistence length can be defined by $\ell_{p}\equiv \langle v\rangle t_{p}\approx 13\;\mu \text{m}$.

There are two features of the correlation function that differ from a pure exponential decay. First, there is a sharp decrease in correlations over the smallest time interval, indicating that there is a short-time decoherence in cellular motions. This could result from various factors such as small fluctuations in cell centers due to cell shape changes, short-time diffusive-like motions of cells, and experimental noise. Second, the correlation function *C*(*t*_1_, *t*_2_;*τ*) is nearly constant at late times, possibly indicating that a small percentage of cells move persistently for very long times. Any model designed to fully describe the behavior of T cells should take these factors into consideration.

#### Modeling cell migration

The data presented in Fig. 3 provide strict constraints that a proposed model for CD8^+^ T cell migration must satisfy. Considering these factors together, a variety of multimodal persistent random walks and generalized Lévy walks were tested. Some of these models could generate the observed correlation function and/or the MSD, but none of the models tested could produce the displacement distributions in Fig. 3B (*e.g.*, the run/pause model proposed in [21] can reproduce the MSD, but not the displacement distributions).

The model that describes all aspects of Fig. 3 was realized through the observation that each of the displacement distributions in Fig. 3B can be fit reasonably well using a double Gaussian fitting function, *Q*_Δ*t*_(*r*):

$$Q_{\Delta t}(r)=\frac{c_{1}}{\sqrt{4\pi D_{1}\Delta t}}e^{-\frac{r^{2}}{4D_{1}\Delta t}}+\frac{c_{2}}{\sqrt{4\pi D_{2}\Delta t}}e^{-\frac{r^{2}}{4D_{2}\Delta t}}.$$ (3)

The fit parameters are the motility parameters, *D*_1_ and *D*_2_ and the weights, *c*_1_ and *c*_2_, of each of the Gaussians, where *c*_1_ + *c*_2_ = 1 for normalization of the entire probability distribution. The fit of Eq. 3 to the cell displacement distributions, *P*_Δ*t*_(*r*), at various times was found using Mathematica (Wolfram Research, Inc., Champaign, IL). All probability distributions, *P*_Δ*t*_(*r*), in Fig. 3B are well-described by *c*_1_ = 0.3, *c*_2_ = 0.7, *D*_1_ = 0.2 *μ*m^2^/min, and *D*_2_ = 9 *μ*m^2^/min.

The fact that the displacement distributions could be fit by a double Gaussian suggests a model with two distinct populations. Therefore we constructed a model in which one population, comprising *c*_1_ = 30% of all walkers, migrates via Brownian walk with a three-dimensional motility coefficient of $D_{1}^{\left(3D\right)}=3D_{1}=0.6\;\mu {\text{m}}^{\text{2}}\text{/min}$. The other *c*_2_ = 70% of walkers migrates through a persistent random walk. This second population is required for obtaining the long tails of the probability distributions, *P*(*r*(*t*)), and its persistence is required for the exponential decay of the correlation function, *C*(*t*_1_, *t*_2_;*τ*). We made the simplifying assumption that persistent walkers have the same average velocity 〈*v*〉 = 6.6 *μ*m/min, the value measured by our experiments. The actual persistence time, *τ*_*p*_ = 3.5 min (≠ *t*_*p*_, the apparent persistence time), for these walkers was determined through the relation $D_{2}^{\left(3D\right)}={\langle v\rangle }^{2}\tau_{p}/6$. In this model, these walkers move at a velocity, *v*, drawn from an exponential distribution with mean 〈*v*〉 for a time, *t*, drawn from an exponential distribution of mean *τ*_*p*_. At the end of a run, the walker chooses a new *v*, *t*, and migration direction. This additional randomness adds variability to our model to more accurately mimic experimental data. Moreover, drawing speeds, *v*, from an exponential distribution mimics the observed instantaneous speed distribution, *P*(*v*), which decays exponentially for large speeds (Fig. 3E). Finally, in order to qualitatively capture the short-time decorrelation of cell motions, the second population of walkers also experiences a small amount of Brownian noise with diffusion coefficient *D*_noise_ = 0.6 *μ*m^2^/min.

In these simulations, walkers were modeled in a finite imaging volume with dimensions 500 *μ*m × 500 *μ*m × 68 *μ*m. Walkers could both enter and leave the field of view. As in the actual experiments, a walker only contributes to the migration statistics during the time interval in which it is in the imaging volume, which is typically a few minutes. Consequently, this model should reflect many of the limitations inherent in the actual experiment.

Indeed, the model reproduces all of the statistical measures shown in Fig. 3 reasonably well and simulated trajectories (Fig. 3F) are qualitatively similar to experimentally observed cell motions (Fig. 3G). The track moment of inertia eigenvalue distribution (dashed lines in Fig. 3A), walker displacement distributions (solid lines in Fig. 3B), and MSD (line in inset to 3C) closely match the experimentally observed statistical measures. The agreement between the model and data explains why the scaled distributions $\tilde{P}(\rho )$ (inset to Fig. 3B) do not collapse; clearly, a double Gaussian distribution cannot be fully characterized by a single time-dependent length scale, *ζ*(*t*). In addition, while the model does not precisely reproduce the correlation function, it captures the main qualitative features (line in Fig. 3D): marked decorrelation over short times and decay with an *apparent* persistence time of *t*_*p*_ ≈ 2 min. Other models for T cell migration neither quantitatively nor qualitatively reproduce these correlations (S1 Fig.). It is likely that a more complicated model could more accurately reproduce the correlation function. Such a model might include, for instance, features such as finely-tuned short-time decorrelation, a small third population of extremely persistent walkers, and cells that switch populations. However, our model has the advantage of describing the data while retaining relative simplicity.

## Discussion

The last decade has seen tremendous advances in the ability to image the behavior of lymphocyte populations [3], but there are several important limitations to imaging experiments and data analyses of cell tracks that have hindered efforts to interpret cell migration quantitatively. For example, the finite imaging volume and *z*-depth of experiments can skew the interpretation of migration data, and the effects of the finite image volume negate any advantage gained by imaging cell populations for longer times. Due to the effects of the shallow depth of the imaging volume, there are severe truncation effects due to cells prematurely leaving the field of view. It is important to recognize that these effects cannot be remedied by simply imaging over a longer time interval; instead, improvements require experiments with greater imaging volume and thus increased *z*-depth. Less obvious effects such as global drift can further obscure the true nature of cell migration; this may be mitigated by adjusting tracks according to the motion of auto-fluorescent particles. These experimental issues necessitate a comprehensive analysis that goes beyond standard measures such as the mean-squared displacement and meandering index.

In addition, several tests for anisotropy and directionality are required because different measurements capture different types of anisotropic behavior. To directly analyze migration statistics, one should construct probability distributions of displacements over various time intervals and measure correlations in cell trajectories. Even without experimental artifacts such as the limited field of view, common measures such as the mean-squared displacement are not sufficient to distinguish details of cell migratory behavior without further information. For instance, a major drawback of the MSD analysis is that different models may produce identical MSD curves, yet differ in a variety of key aspects including mean velocity or persistence time. We have presented quantitative methods to minimize and account for these limitations, and quantitatively describe cell migration. Together, these techniques minimize errors due to drift (Fig. 1), reliably detect anisotropic cell migration (Fig. 2), and provide a strong connection between migration models and experimental data (Fig. 3).

With these methods, we have found that the migration of CD8^+^ T cells in lymph nodes in the absence of inflammation is reasonably well-described by a model with two distinct populations of stochastic walkers (Fig. 3). The first population migrates by a pure Brownian walk. The other population, comprising most of the walkers, migrates by a persistent random walk and is subject to a small amount of Brownian noise. This model can also be interpreted as the aggregate of a paused population and an active population with a small amount of overall noise.

While this model has some similarities with existing models, it differs from previous models for CD8^+^ T cell migration in the lymph node, which describe cells as a single, homogeneous population of persistent random walkers [5, 19–22]. Our model explicitly incorporates heterogeneity in the CD8^+^ T cell population. Furthermore, in contrast to previous run/pause models for T cells in the lymph node [21], runs and pauses are not well-mixed. Instead, in our refined model, cells that are essentially paused except for slow Brownian-like motion, remain in the paused state for many minutes at a time (the entire duration), and similarly, cells migrating by persistent random walks move continuously for at least ten minutes. This model, in contrast to existing models and common practice [1, 41–43], does not choose an arbitrary speed cutoff below which cells are assumed to be paused and data is discarded. Most importantly, this model successfully describes multiple cell migratory statistical measures (Fig. 3), rather than just the MSD.

There are a variety of possible explanations for the observed heterogeneity in migratory behavior. For instance, in these experiments, CD8^+^ T cells were imaged throughout the lymph node, and thus, likely in multiple zones within the lymph node. Thus, it is possible that the two populations in the model represent migration in distinct regions of the lymph node. Alternatively, previous studies using this experimental system have shown that this population of OT-I CD8^+^ T cells express variable levels of the chemokine receptors CCR5 and CCR7 [24], which could impact migration. Finally, it is unlikely that the paused population of cells arises from an experimental artifact such as phototoxicity. Indeed, paused cells are alive since they exhibit shape fluctuations and even, after very long time scales, begin to migrate.

Interestingly, T cells in uninflamed lymph nodes do not migrate via generalized Lévy walks, as activated T cells do in the brain during chronic toxoplasmosis [6]. While generalized Lévy walks may enable T cells to efficiently find rare target parasites [6], the long runs in the Lévy walk may be less beneficial for T cells that must frequently interact with dendritic cells. The observed differences in migration statistics may be indicative of the cell extrinsic or intrinsic differences between the two cell populations. For instance, it is likely that the structural features within these tissues, which may act as a scaffold for cell crawling [1, 21, 44], are different in the two tissues. In addition, the T cell population in lymph nodes in the steady state is not as activated as the cells in the brain, which could also affect migratory behavior. Together, these observations suggest that CD8^+^ T cell populations with distinct functions migrate differently.

While the model is successful in characterizing many aspects of cell migration (Fig. 3), there are deviations from measured cell statistics. These differences reflect the difficulty in systematically identifying a simple model that accurately and comprehensively describes walk statistics. Both fluctuations within individual cells and variations within cell populations complicate the overall behavior and ensuing analysis. This problem may be mitigated by accumulating better statistics; for example, if individual cells could be followed for hours throughout the entire lymph node, instead of minutes in a small volume, the whole-population analysis could instead be carried out for individual cell tracks. Additionally, further improvements to analytical and computational methods will lead to more accurate cell migration modeling. However, while various measures and techniques to understand and describe migratory behavior have been developed [4–6, 15, 36, 45–49], less has been done to systematically build robust migration models. The methodology described in this paper, combined with generalizations of new techniques for analyzing heterogeneous migration statistics [36, 46, 48, 49], could achieve this goal.

In general, use of these statistical approaches require relatively large amounts of data (more than 100 cell tracks) and numerical simulations of random walk models. Despite these difficulties, these methods can provide powerful insights into cell migratory behavior, and they will be useful for characterizing migration in future studies. In turn, developing these more accurate models will help connect cell migration to immune function and lead to a deeper understanding of immune response.

## Methods

### Ethics statement

All procedures involving mice were reviewed and approved by the Institutional Animal Care and Use Committee of the University of Pennsylvania (Animal Welfare Assurance Reference Number #A3079–01) and were in accordance with the guidelines set forth in the Guide for the Care and Use of Laboratory Animals of the National Institute of Health.

### Isolation of fluorescent CD8^+^ T cells

CD8^+^ T cells were isolated as previously described [24]. Briefly, cells were isolated from the spleen and peripheral lymph nodes of DPE-GFP OT-I transgenic mice (OT-I^GFP^). Single cell suspensions were obtained by mechanical homogenization. Red blood cells were removed by hypotonic lysis. T cells were purified using the mouse T cell enrichment columns (R&D systems, Minneapolis, MN). 2–5 × 10^6^ purified OT-I^GFP^ cells were injected into recipient mice intravenously.

### Multi-photon microscopy

Mice were euthanized by CO_2_ asphyxiation 16–24 hours following T cell transfer. The mesenteric lymph nodes were removed immediately, with minimal mechanical disruption. The lymph nodes were immobilized in 1% agarose in a heated chamber where specimens were constantly perfused with warmed (37°C), oxygenated media (phenol-red free RPMI 1640 supplemented with 10% FBS, Gibco). The temperature in the imaging chamber was maintained at 37°C using heating elements and monitored using a temperature control probe. Imaging was performed using a Leica SP5 multi-photon microscope system (Leica Microsystems, Mannheim, Germany) equipped with a resonant scanner, picosecond laser (Coherent Inc., Santa Clara, CA), and external detectors that allow simultaneous detection of emissions of different wavelengths. Enhanced GFP was excited using laser light of 920 nm. Images were obtained using a 20X water-dipping lens. Four-dimensional imaging data was collected by obtaining images from the *x*-, *y*-, and *z*-planes, with a *z*-thickness of 68 *μ*m and step size of 4 *μ*m to allow for the capture of a complete z-series every 20 seconds for period of 15 minutes. 8 separate image series were taken. Individual cells were tracked using Volocity software (PerkinElmer, Waltham, MA), giving the *x*-, *y*-, and *z*-coordinates of each cell at every time point.

### Numerical simulations

We implement standard Brownian dynamics algorithms for numerical walker models [50]. For each walker, we draw a run time, *t*, and speed, $\left|\vec{v}\right|$, from distributions using pseudorandom number generators. A direction for the velocity vector $\vec{v}$ is also uniformly randomly chosen. At each time step, we add $\vec{v}\delta t$ to the walker position. We use a time step of *δt* = 0.001 s. When the run time, *t*, has passed, we randomly draw new run times, speeds, and directions. Diffusive noise is added by adding $(6D\delta t)\hat{r}$ to the walker position at each time step, where $\hat{r}$ is a unit vector that points in a random direction. We simulate 5,000 walkers in a 600 *μ*m × 600 *μ*m × 170 *μ*m volume, but only collect data on walkers if they are within a particular 500 *μ*m × 500 *μ*m × 68 *μ*m “imaging” volume. Prior to data collection, we simulate walkers for a short period of time (typically a minute) in order to avoid artifacts at early times.

### The moment of inertia tensor

The 2D moment of inertia tensor, **I**, is given by [31, 32]:

$$I=\left(\begin{array}{cc} I_{xx} & I_{xy}\\ I_{yx} & I_{yy} \end{array}\right),\;I_{xx}=\sum_{i}^{N}y_{i}^{2},\;I_{xy}=I_{yx}=-\sum_{i}^{N}x_{i}y_{i},\;I_{yy}=\sum_{i}^{N}x_{i}^{2}.$$ (4)

*I*_*xx*_, *I*_*xy*_, *I*_*yx*_, and *I*_*yy*_ are summations of products of displacements, *x*_*i*_ and *y*_*i*_. For the average moment of inertia tensor, $\bar{I}$, the summation runs over all cellular motions, so that *i* indexes individual steps and *N* is the total number of steps. For individual track tensors, **I**_*n*_, the summation is over all displacements for the individual cell, so while *i* still indexes individual displacements, *N* is now the number of frames for the track. An inertia tensor, **I**_*n*_, can be calculated for each track, but only tracks of the same length (*e.g.,* complete tracks) should be averaged together. In order to find the eigenvalues of **I**, follow standard linear algebra methods [31, 32]. The eigenvalues are found by the solving det(I−λ1)=0 so that $\lambda_{\pm }=I_{xx}+I_{yy}\pm \sqrt{I_{xx}^{2}-2I_{xx}I_{yy}+I_{yy}^{2}+4I_{xy}^{2}}$, where *λ*_1_ = *λ*_+_ and *λ*_2_ = *λ*_−_. Finally, note that for the asphericity calculation, one typically takes the moment of inertia tensor about the center of the track [33]; to do this replace *x*_*i*_ and *y*_*i*_ in Eq. 4 with $x_{i}-\bar{x}$ and $y_{i}-\bar{y}$, respectively, where $\bar{x}=\frac{1}{N}\sum_{i}^{N}x_{i}$ and $\bar{y}=\frac{1}{N}\sum_{i}^{N}y_{i}$.

## Supporting Information

S1 Fig Comparison of displacement correlations of different models to experimental data. None of the models tested (lines) provide a quantitative description of experimentally observed T cell displacement correlations (circles). However, the two population model proposed in the main text (solid line) provides the best qualitative description of all the models. Other models for T cell migration in lymph nodes, such as a single population of persistent random walkers (dashed line) and a single population of persistent random walkers that run and pause (dotted line) provide a poor qualitative and quantitative description of correlation data.
(PDF)
