# Supplementary figures and images for: Heterogeneous CD8+ T Cell Migration in the Lymph Node in the Absence of Inflammation Revealed by Quantitative Migration Analysis
Source: PLoS Comput Biol. 2015 Feb 18;11(2):e1004058. doi: 10.1371/journal.pcbi.1004058 (PMC4334969; doi:10.1371/journal.pcbi.1004058)

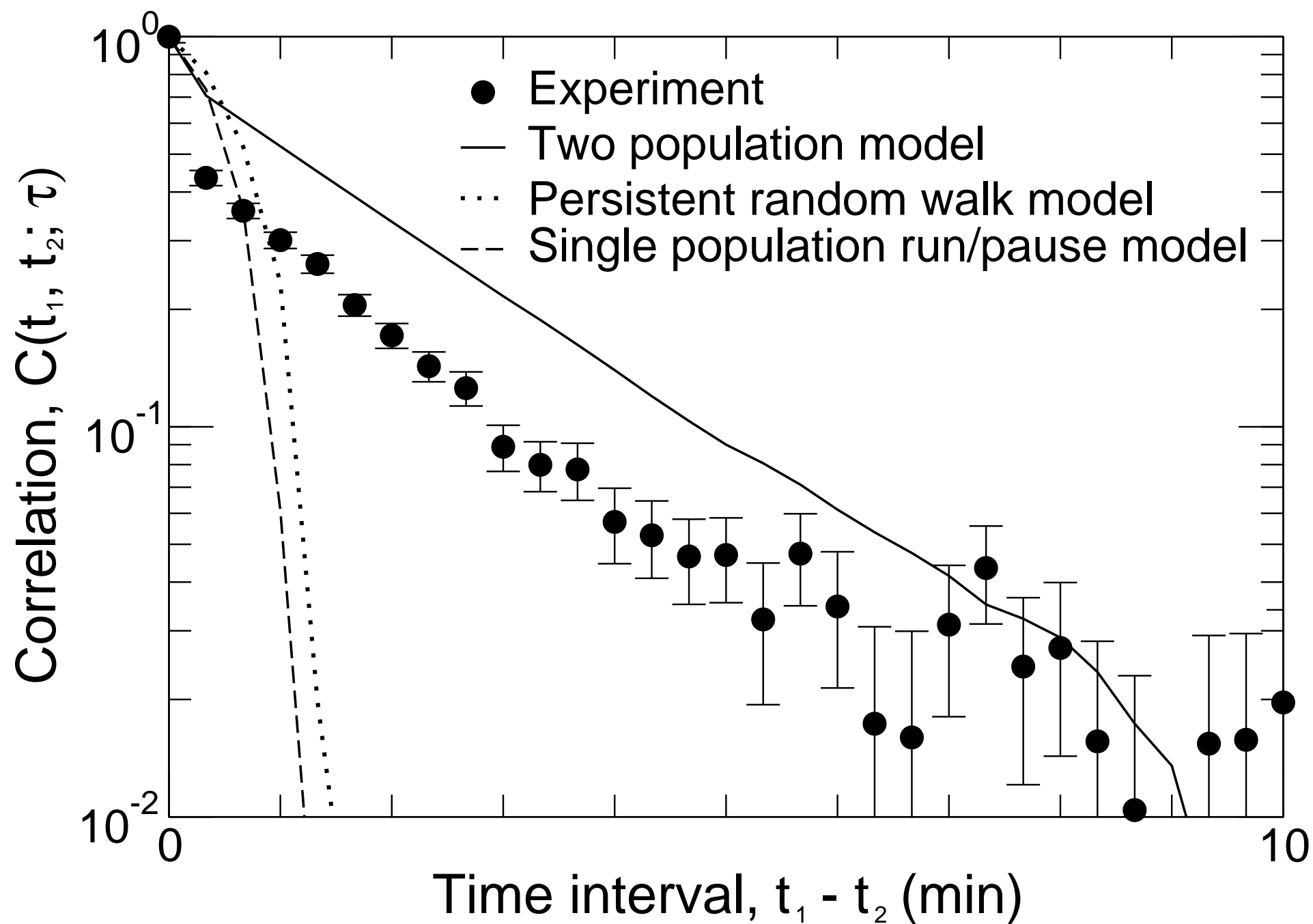

Supplement: S1 Fig — None of the models tested (lines) provide a quantitative description of experimentally observed T cell displacement correlations (circles). However, the two population model proposed in the main text (solid line) provides the best qualitative description of all the models. Other models for T cell migration in lymph nodes, such as a single population of persistent random walkers (dashed line) and a single population of persistent random walkers that run and pause (dotted line) provide a poor qualitative and quantitative description of correlation data. (PDF) [file pcbi.1004058.s001.pdf]
